# Supplementary material for: Direct observation of interdependent and hierarchical kinetochore assembly on individual centromeres
Source: Nucleic Acids Res. 2025 Oct 15;53(19):gkaf1038. doi: 10.1093/nar/gkaf1038 (PMC12526914; doi:10.1093/nar/gkaf1038)
Supplement: gkaf1038_Supplemental_Files [file gkaf1038_supplemental_files.zip › 9.11.25 Supplemental figures_R1.docx]

**
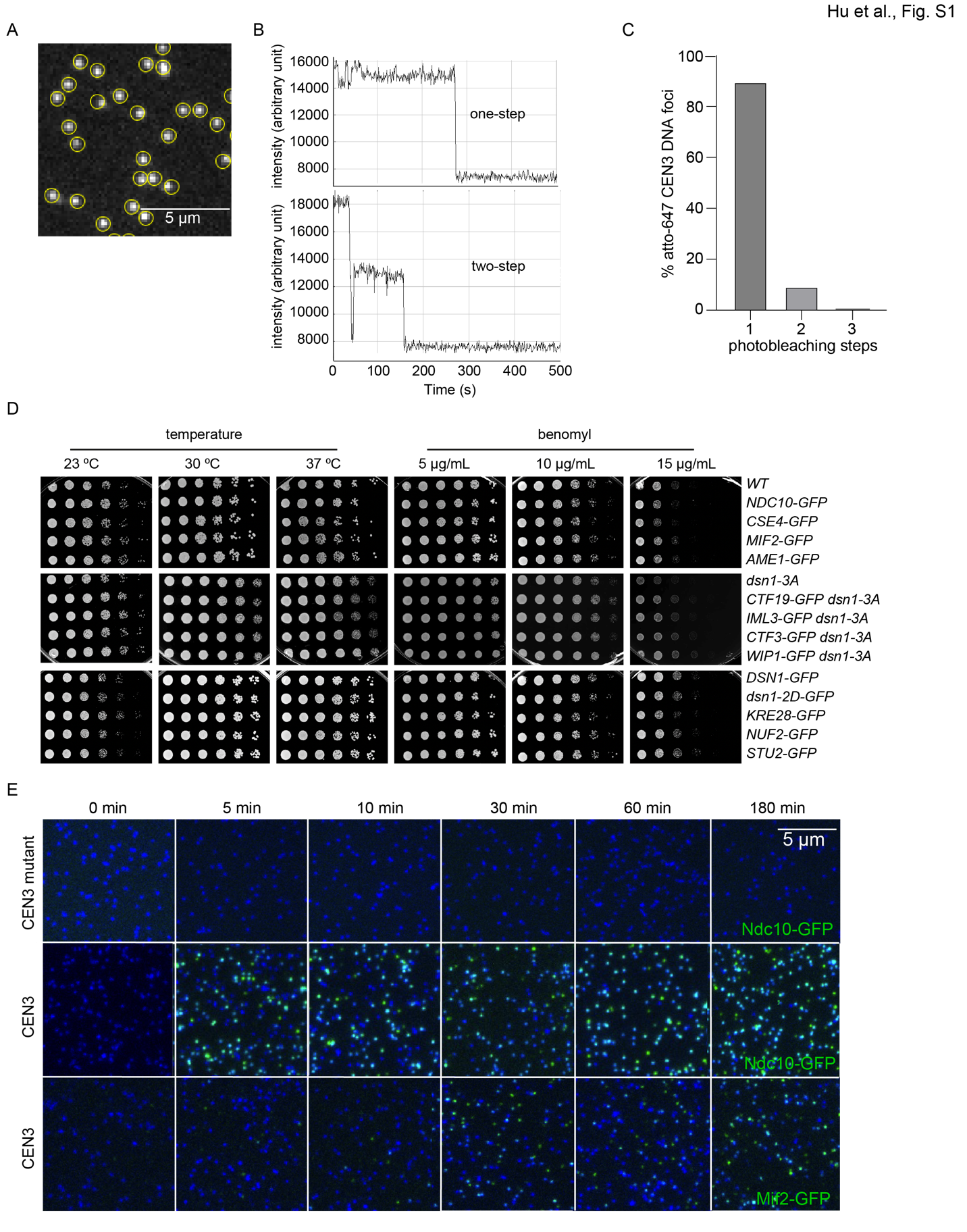
**

**Supplementary Figure S1**. **GFP-tagged kinetochore proteins bind specifically to CEN3 DNAs.**

1. Representative image of CEN3 DNAs. Yellow circles indicate the automatic selection of each DNA focus detected by fluorescence (Atto-647).
2. Representative Atto-647 fluorescence intensity trace over time showing one-step (top) and two-step photobleaching (bottom).
3. Quantification of photobleaching steps for each DNA focus from panel (A) analyzed in MATLAB using previously a described step-finding algorithm (27).
4. Five-fold serial dilutions of yeast strains containing the indicated GFP tags. Strains include *NDC10-GFP* (SBY22903),*CSE4-GFP* (SBY22195), *MIF2-GFP* (SBY22094), *AME1-GFP* (SBY22119), *dsn1-3A* (SBY14171), *CTF19-GFP* *dsn1-3A* (SBY24416), *IML3-GFP dsn1-3A* (SBY24442), *CTF3 dsn1-3A* (SBY24432), *WIP1-GFP dsn1-3A* (SBY24440), *DSN1-GFP* (SBY22153), *dsn1-2D-GFP* (SBY22159), *KRE28-GFP* (SBY24188), *NUF2-GFP* (SBY23256), *STU2-GFP* (SBY22135), and the untagged parental control (wild type, SBY4). Cells were plated on YPD or benomyl media. Cells were grown on YPD at the indicated temperatures for 48 hours or at the indicated benomyl concentrations at 23 ºC.
5.
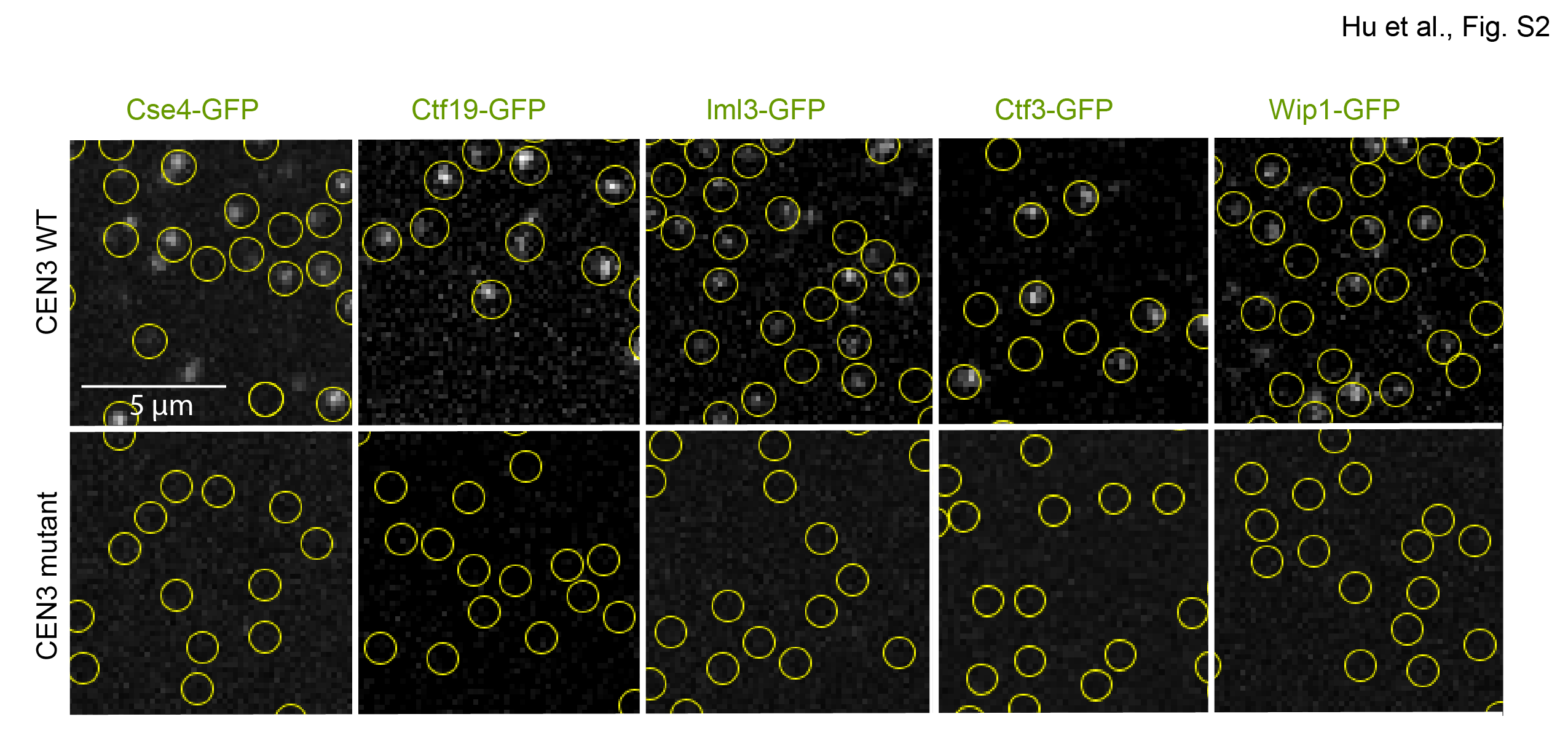
Representative TIRFM images of Mif2-GFP (SBY22094) and Ndc10-GFP (SBY22903) (green foci) after a time series incubation with CEN3 DNAs (blue foci, middle and bottom rows) and CEN3 mutant DNAs (blue foci, top row). Yeast were arrested in mitosis using benomyl prior to harvesting. The lysates were washed off before imaging.

**Supplementary Figure S2**. **GFP tagged kinetochore proteins bind specifically to CEN3 DNAs.**

Representative images of CEN3 or CEN3 mutant DNAs (at locations indicated by yellow circles) after kinetochore assembly in lysates made from strains with GFP-tagged Cse4 (SBY22195), Ctf19 (SBY22116), Iml3 (SBY22199), Ctf3 (SBY22203), and Wip1 (SBY22207). Yeast cells were arrested in mitosis with benomyl prior to harvesting the lysates. The lysates were washed off the slides after 180 minutes of incubation at room temperature (21-23 ºC) before imaging.

**
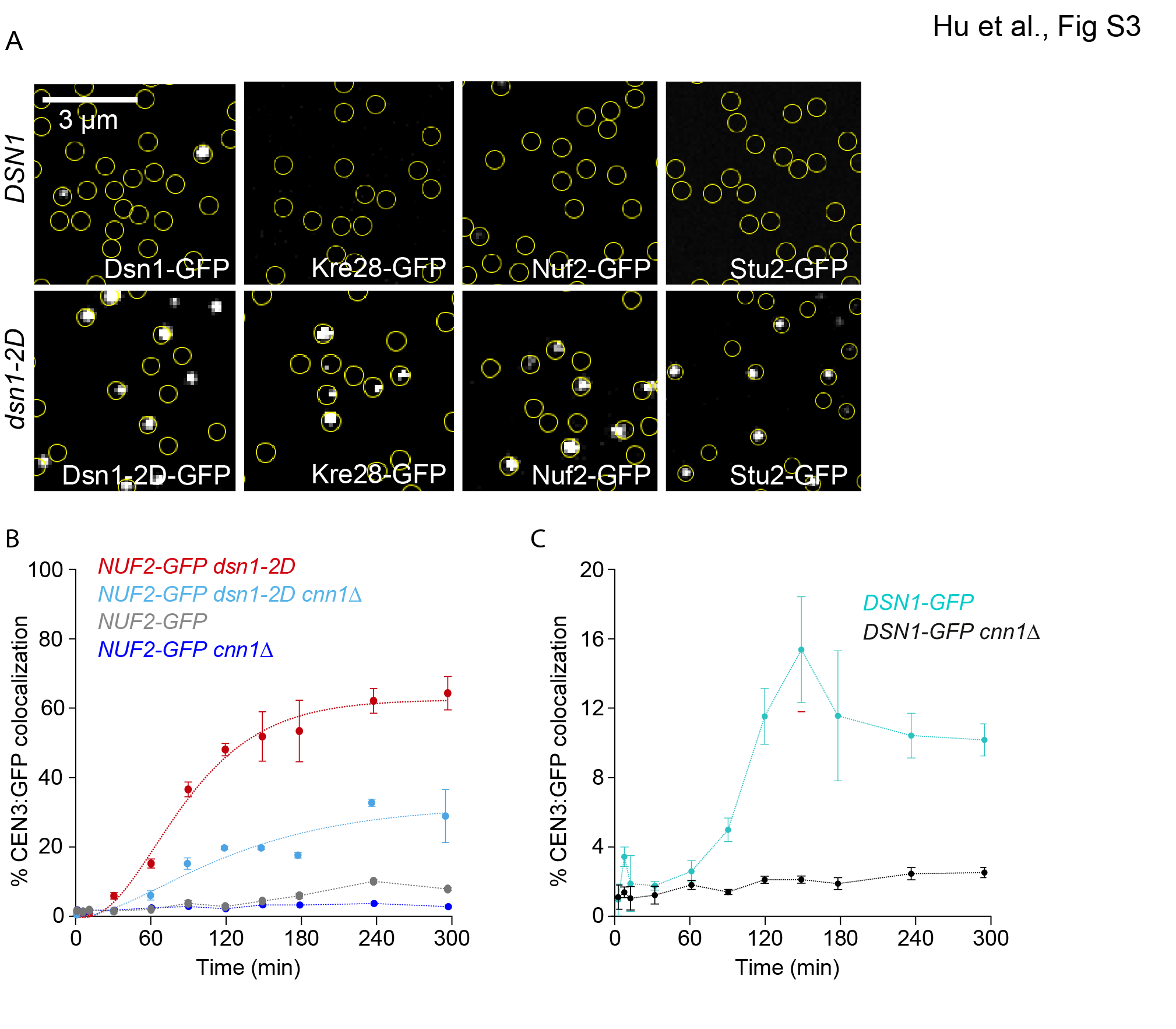
**

**Supplementary Figure S3. *dsn1-2D* promotes outer kinetochore assembly.**

1. Representative TIRFM images of GFP-tagged outer kinetochore proteins (grayscale) after 180 min incubation of CEN3 DNAs (yellow circles) with indicated lysates. Dsn1-GFP, Nuf2-GFP, Kre28-GFP, and Stu2-GFP are shown in the *DSN1* background (top; using lysates from strains SBY22153, SBY23256, SBY24188, and SBY22135, respectively) or in the *dsn1-2D* background (bottom; using strains SBY22159, SBY23258, SBY24190, SBY22133, respectively).
2. Percentages of CEN3 DNAs with colocalized Nuf2-GFP in *dsn1-2D* (SBY23258, data replotted from Figure 3D), *dsn1-2D* *cnn1∆* (SBY24414), *DSN1* (SBY23256, same data from 3A), and *DSN1 cnn1∆* (SBY24412) lysates.
3. Percentages of CEN3 DNAs with colocalized Dsn1-GFP for strains *DSN1-GFP* (SBY22153, same data as Figure 3A) and *DSN1-GFP cnn1∆* (SBY24769).

All error bars represent the standard deviation over three biological repeats. At least 3,000 DNA molecules were imaged for each time point from each biological replicate.

**
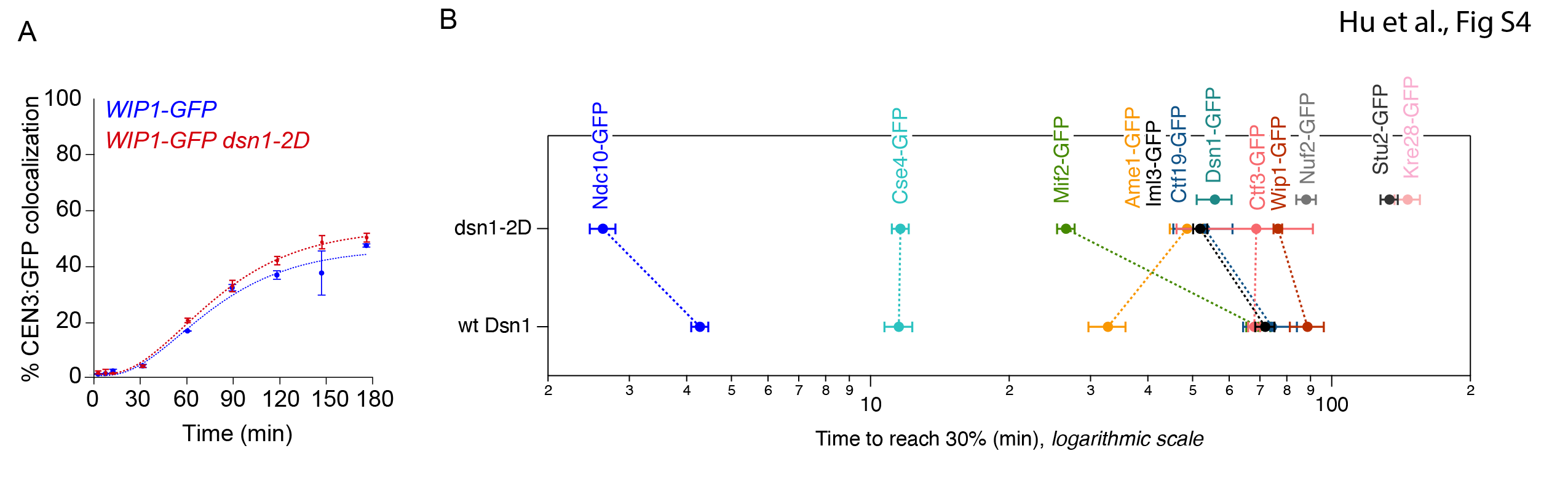
**

**Supplemental Figure S4.** T_30_ varies between wild type and *dsn1-2D* strains.

1. Percentage of CEN3 DNA with colocalized Wip1-GFP in *DSN1* (SBY22207, data replotted from Figure 2A) and *dsn1-2D* (SBY22205) strains.
2. Direct comparison of *T_30_*  between wild type and *dsn1-2D* strains. Numbers were obtained from Supplemental table S3.

**
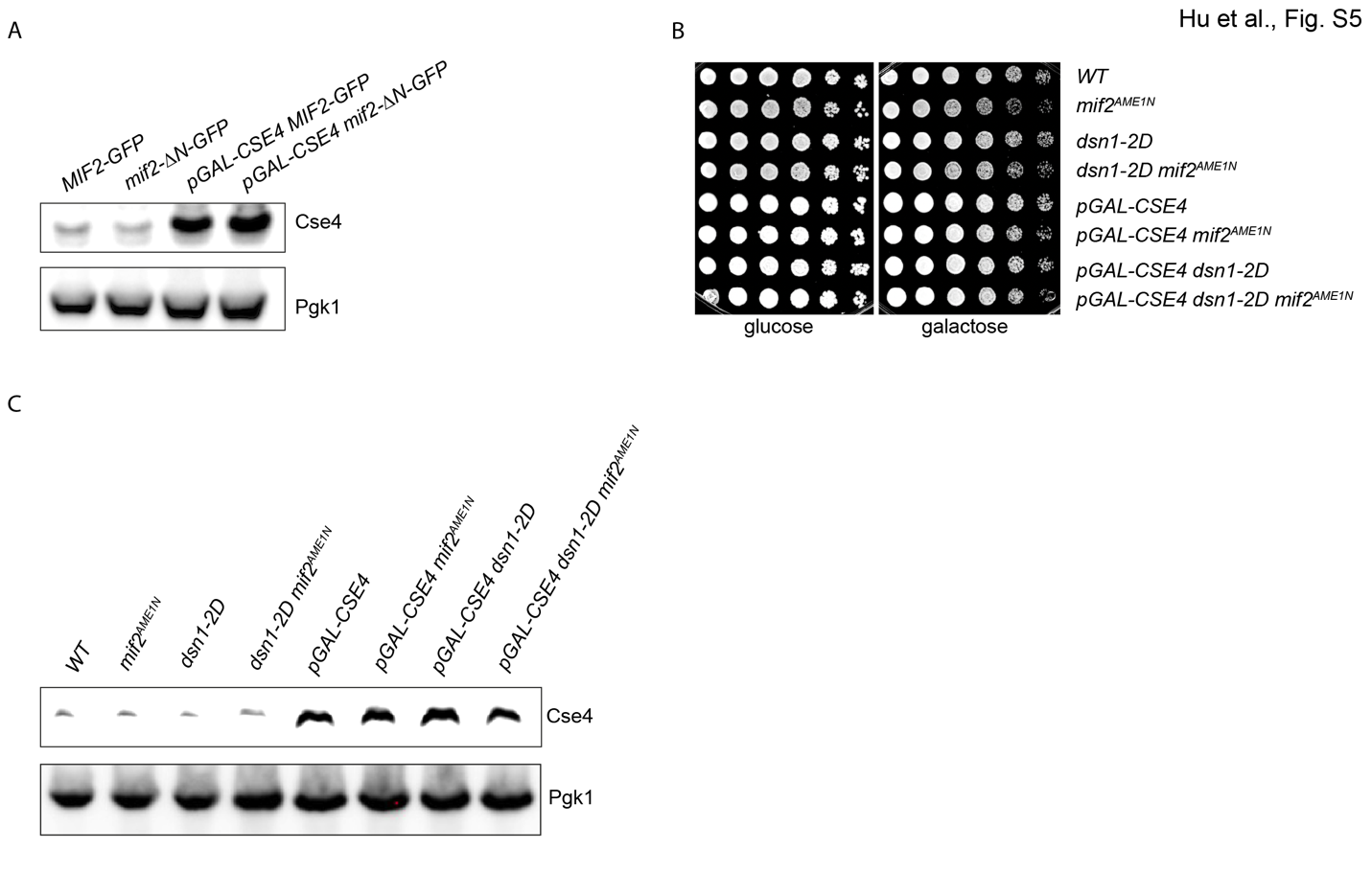
**

**Supplemental Figure S5. *Mif2-AME1N* and *dsn1-2D* do not alter cell growth in the presence or absence of Cse4 overexpression.**

1. Immunoblotting with anti-Cse4 and anti-Pgk1 antibodies indicates that the levels of wild type and overexpressed Cse4 are similar in MIF2 and mif2-∆N strains. Pgk1 is used as a loading control. Yeast lysates were prepared from *MIF2-GFP* (SBY22095), *mif2-∆N* (SBY23249), *pGAL-CSE4 MIF2-GFP* (SBY24220), and *pGAL-CSE4 mif2-∆N-GFP* (SBY24222) yeast strains grown in galactose.
2. Five-fold serial dilutions of yeast strains containing the indicated mutations. Strains include wild type (SBY3), *mif2^AME1N^* (SBY23570), *dsn1-2D* (SBY14150), *dsn1-2D mif2^AME1N^* (SBY23255), *pGAL-CSE4* (SBY24210), *pGAL-CSE4 mif2^AME1N^* (SBY24779), *pGAL-CSE4 dsn1-2D* (SBY24763), and *pGAL-CSE4 dsn1-2D mif2^AME1N^* (SBY24764).
3. Immunoblotting with anti-Cse4 and anti-Pgk1 antibodies indicates that the levels of wild type and overexpressed Cse4 are similar between strains. Yeast lysates were prepared from SBY3, *mif2^AME1N^* (SBY23570), *dsn1-2D* (SBY14150), *dsn1-2D mif2^AME1N^* (SBY23255), *pGAL-CSE4* (SBY24210), *pGAL-CSE4 mif2^AME1N^* (SBY24779), *pGAL-CSE4 dsn1-2D* (SBY24763), and *pGAL-CSE4 dsn1-2D mif2^AME1N^* (SBY24764) strains grown in galactose. Pgk1 is used as a loading control.


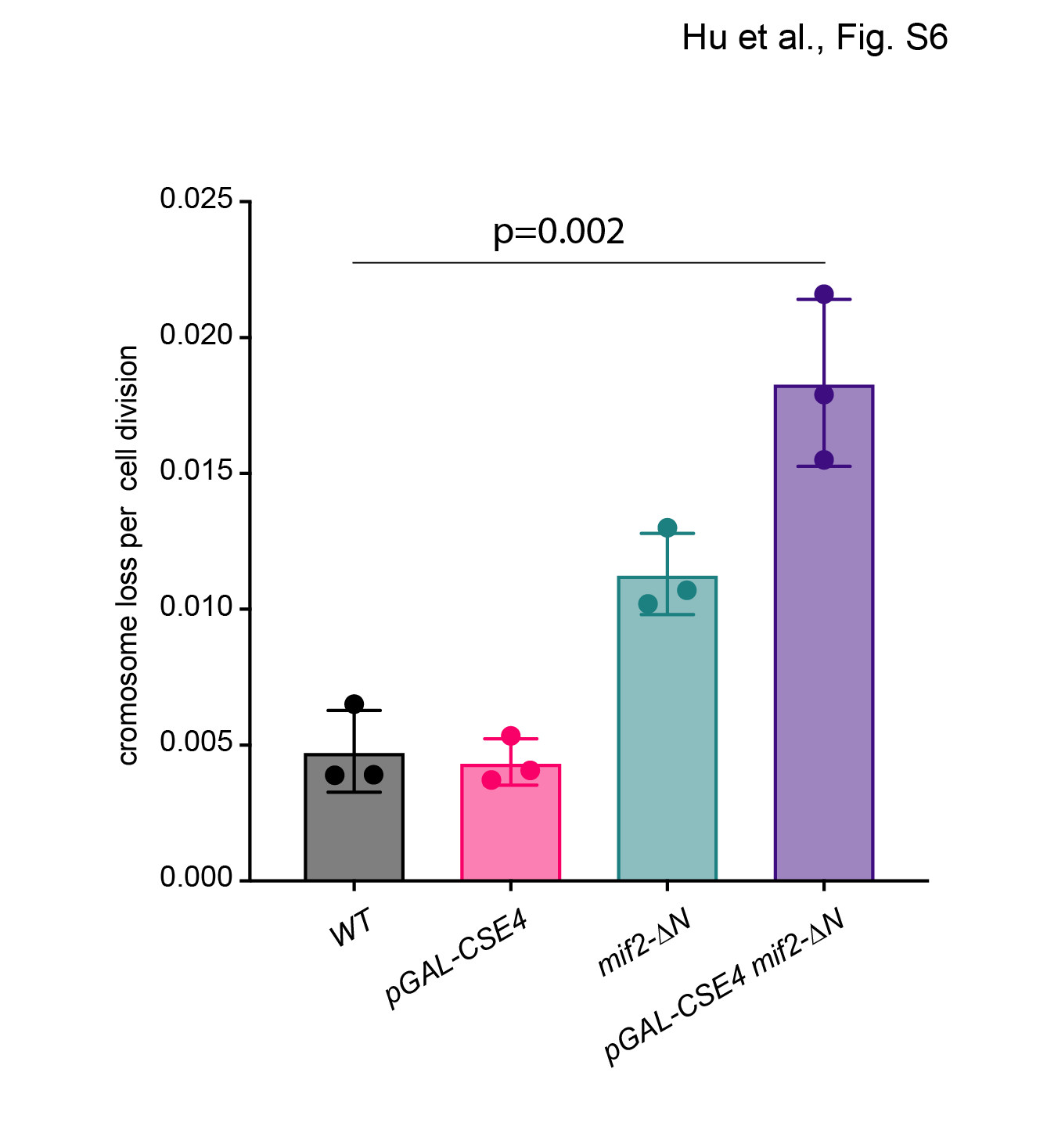


Supplemental Figure S6. *mif2-∆N* increases mini-chromosome loss rate in cells with overexpressed Cse4.

Mini-chromosome loss assay measured by flow cytometry in wildtype (SB24751), *pGAL-CSE4* (SB24752), *mif2-∆N* (SB24753), and *pGAL-CSE4 mif2-∆N* (SB24754). Chromosome loss rate calculated based on Zhu et al. (29). Note that there are GFP tagged kinetochore proteins which might create an artificially higher baseline loss rate in all strains.
